# The autophagy-related genes *BbATG1* and *BbATG8* have different functions in differentiation, stress resistance and virulence of mycopathogen *Beauveria bassiana*

**DOI:** 10.1038/srep26376

**Published:** 2016-05-20

**Authors:** Sheng-Hua Ying, Jing Liu, Xin-Ling Chu, Xue-Qin Xie, Ming-Guang Feng

**Affiliations:** 1 Institute of Microbiology, College of Life Sciences, Zhejiang University, Hangzhou, Zhejiang, 310058, People’s Republic of China

## Abstract

Autophagy-related proteins play significantly different roles in eukaryotes. In the entomopathogenic fungus *Beauveria bassiana*, autophagy is associated with fungal growth and development. *BbATG1* (a serine/threonine protein kinase) and *BbATG8* (a ubiquitin-like protein) have similar roles in autophagy, but different roles in other processes. Disruption mutants of *BbATG1* and *BbATG8* had impaired conidial germination under starvation stress. The mutant Δ*BbATG8* exhibited enhanced sensitivity to oxidative stress, while a Δ*BbATG1* mutant did not. *BbATG1* and *BbATG8* showed different roles in spore differentiation. The blastospore yield was reduced by 70% and 92% in Δ*BbATG1* and Δ*BbATG8* mutants, respectively, and the double mutant had a reduction of 95%. Conidial yield was reduced by approximately 90% and 50% in Δ*BbATG1* and Δ*BbATG8* mutants, respectively. A double mutant had a reduction similar to Δ*BbATG1*. Additionally, both *BbATG1* and *BbATG8* affected the levels of conidial protein BbCP15p required for conidiation. The virulence of each autophagy-deficient mutant was considerably weakened as indicated in topical and intrahemocoel injection assays, and showed a greater reduction in topical infection. However, *BbATG1* and *BbATG8* had different effects on fungal virulence. Our data indicate that these autophagy-related proteins have different functions in fungal stress response, asexual development and virulence.

Autophagy (macroautophagy) is an evolutionally conserved self-degradation process found in all eukaryotes^1^,^2^, and has now been linked to many developmental processes in filamentous fungi^3^,^4^,^5^,^6^,^7^. Currently, increasing evidence indicates that the autophagy-related genes (ATG) of animals and yeasts are associated with a variety of non-autophagic processes such as secretion, signal transduction, and membrane reorganization^8^,^9^.

In yeast, 40 autophagy-related genes have been identified and functionally characterized. Of these, 18 ‘core’ machinery genes (e.g., *ATG1* and *ATG8*) are obligatory for the autophagic process^10^ and are also conserved in filamentous fungi (e.g., *Aspergilus nidulans*, *Neurospora crassa* and *Magnapothe grisea*)^11^. ATG1p (a serine/threonine protein kinase) and ATG8p (a ubiquitin-like protein) are indispensable for autophagosome induction and expansion, respectively^5^,^10^. These two genes are essential for autophagy which is not only required for fungal development, but also play an important role in the establishment of infection by fungal pathogens. In *Podospora anserina*, autophagy is associated with cell death. Disruption of *PaATG1* and *PaATG8* suppresses cell death by incompatibility^12^. In *A. oryzae*, deletion of the *ATG1* or *ATG8* ortholog leads to defects in conidiation and conidial germination under nitrogen starvation conditions^13^,^14^. In *A. fumigatus* (a human pathogen), a Δ*AfATG1* mutant showed severely impaired growth under metal-ion deficient conditions^15^. In *M. grisea* (a plant pathogen), a deletion mutant in *MaATG1* or *MaATG8* lacked normal autophagy and appeared to be defective in appressorium development and pathogenicity^16^,^17^. However, more and more studies indicate that the *ATG1* and *ATG8* genes have different roles in filamentous fungi. In *Ustilago maydis* (a plant pathogen), *ATG1* or *ATG8* is also necessary for autophagy, but each gene affects fungal development and fungal pathogenicity to a different degree. For example, a Δ*ATG8* mutant produced very few telliospores, but telliospore production was not affected in a Δ*ATG1* mutant^18^. In an insect pathogen, *Metarhizium robertsii*, the *ATG8* gene regulates appressorium formation while the *ATG1* gene does not^19^. These findings suggest that the *ATG1* and *ATG8* genes have different, non-autophagic roles during fungal development.

*Beauveria bassiana*, a filamentous fungus, was first discovered by Agostinio Bassi in 1835 as an infectious agent in silkworms^20^. This fungus has been widely used for the biological control of insect pests^21^ and as a model entomopathogenic fungus to study the insect immune response^22^. Under natural conditions, *B. bassiana* depends on its conidia (asexual spores) to maintain the infection cycle. Once adhered to the host cuticles, the conidia germinate and invade the host hemocoel via direct penetration of the host cuticles, followed by a dimorphic conversion and propagation in the host hemocoel^23^,^24^. *B. bassiana* overcomes a series of host immune defenses and finally kills the host^22^. Newly born conidia on cadavers initiate subsequent infection cycles^25^. Autophagy has been linked to the development and virulence of *B. bassiana*^26^, but whether the autophagic process or various autophagic genes regulate fungal physiological functions is far from clear.

In this study, we demonstrate that the *ATG1* and *ATG8* genes play similar roles in autophagy, but differently affect non-autophagic functions. We found that *BbATG1* and *BbATG8* are required for autophagy, growth under starvation and spore differentiation (including conidia and blastospores) in *B. bassiana*. Conidia are asexual spores formed under aerobic conditions and blastospores are yeast-like cells produced in a liquid environment^26^. In addition, we demonstrate that *BbATG8*, but not *BbATG1*, is crucial for fungal resistance to oxidative stress. Furthermore, both Δ*BbATG1* and Δ*BbATG8* mutants showed significantly reduced virulence in a cuticle penetration bioassay. In an injection bioassay, the Δ*BbATG8* mutant evidenced a significant decrease in virulence, but the virulence of the Δ*BbATG1* mutant was not greatly affected. More importantly, we characterized a conidial protein (BbCP15) acting as a downstream target of the autophagic genes during conidial development. Taken together, these results expand our understanding of the different roles of autophagic genes in filamentous mycopathogens.

## Results

### Identification and disruption of *ATG1* and *ATG8* homologues in *B. bassiana*

*MgATG1* and *MgATG8* are two well-studied autophagy-related genes of *M. grisea* that were used as queries to search for homologues in the *B. bassiana* genome database^27^. Two single highly related *B. bassiana* genes, BBA_07025 (E-value, 0.0) and BBA_02302 (8e^−060^), were retrieved and designated as *BbATG1* and *BbATG8*, respectively. The *BbATG1* gene encodes a 930-amino acid serine/threonine protein kinase containing a Pkinase domain that is found in all homologues in animals, plants and fungi (see Supplementary Table S1). The *BbATG8* gene encodes a 118-amino acid protein that contains an Atg8 domain that is also conserved in all homologues in fungi, plants and animals (see Supplementary Table S2). Both BbATG1p and BbATG8p (see Supplementary Fig. S1) are closely related to their respective partners from the other two entomopathogenic fungi (*Cordyceps militaris* and *M. anisopliae*), but less so to those of plant and animal groups.

To determine the potential role of each *ATG* gene in *B. bassiana*, we generated appropriate autophagy-deficient strains using a homologous replacement strategy (see Supplementary Fig. S2). The Δ*BbATG1* and Δ*BbATG8* mutants were identified by PCR screening and further verified by Southern blotting. Applying the same strategy, double mutant (Δ*BbATG1*Δ*BbATG8*) strains were constructed by disruption of *BbATG1* in the Δ*BbATG8* mutant strain. In TEM images, autophagic bodies were not seen in the vacuoles of three gene-disruption mutants under nutrient starvation but were found in the wild type and complementation strains (see Supplementary Fig. S3). This suggests that *BbATG1* and *BbATG8* are both necessary for autophagy in *B. bassiana*.

### Autophagy is associated with fungal development and growth

**Figure 1:**
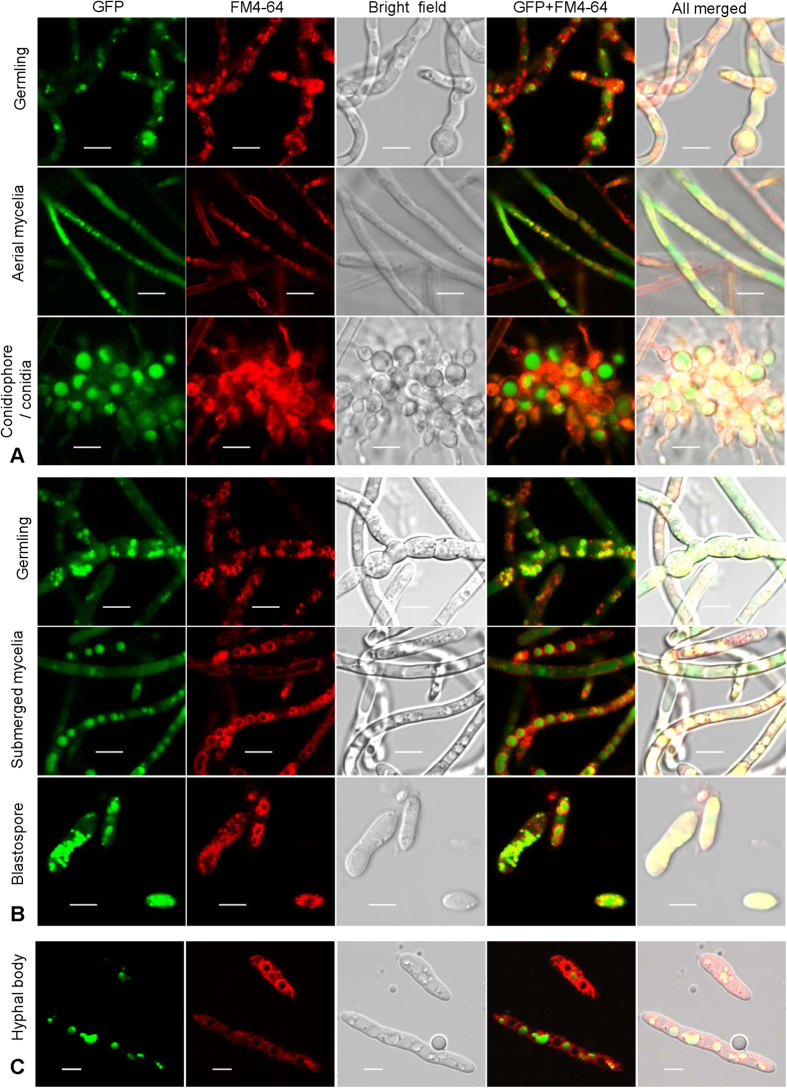
Autophagic process occurs in the entire developmental cycle of *Beauveria bassiana*. A hybrid gene of *eGFP-BbATG8* was integrated in the wild type strain to track the autophagic process in fungal cells at different development stages. Vacuolar membranes were stained by fluorochrome FM4-64. Punctate and globular signals were observed in germilings, mycelia and spores of both surface (**A**) and submerged culture (**B**). In host hemolymph (**C**), the eGFP signals were also clearly observed in hyphal bodies (*in vivo* blastospores). Scale bars, 5 μm.

Localization of ATG8p in cells represents the morphologies of autophagosomes and autophagic bodies, and was revealed by fusing GFP to the N-terminus of ATG8p. Vacuolar membranes were indicated by red fluorescence of fluorochrome FM4-64. Punctate and globular signals of GFP were seen in various cell types such as germlings, mycelia and spores from the surface (Fig. 1A) and submerged cultures (Fig. 1B), respectively, as well as hyphal bodies (a type of yeast-like cell) from the insect hemolymph (Fig. 1C). There was no significant difference in the autophagosome size between aerial and submerged mycelia. Aerial conidia only contained the obvious punctate signals; however, both blastospores and hyphal bodies evidenced additional GFP signal in the vacuoles.

### *BbATG1* and *BbATG8* are required for fungal survival under starvation

**Figure 2:**
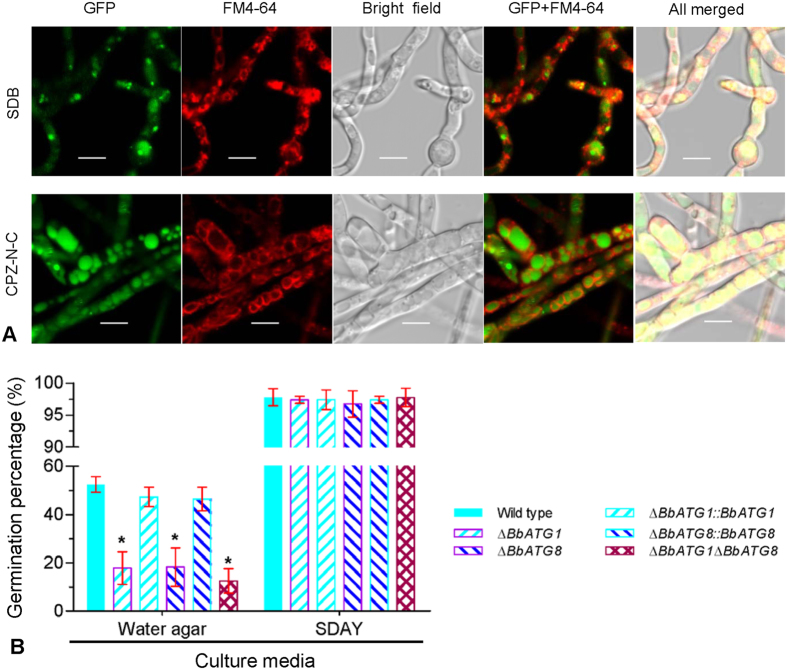
*B. bassiana ATG1* and *ATG8* genes are associated with autophagic roles under starvation stress. (**A**) Cellular localization of eGFP tagged BbATG8p in mycelia under the stress of nutrient deprivation. The GA8 strain expressing *eGFP-BbATG8* was grown in SDB medium for 2 d, and then transferred into CPZ medium without carbon and nitrogen (CPZ-N-C). After a 3-h incubation, almost all eGFP signals appeared in vacuoles. (**B**) Germination percentage of conidia from the various strains. Water agar plates were used to examine the conidial germination under the nutrient-limiting condition, using SDAY plate as the control of rich nutrients. Scale bars, 5 μm.

Autophagy contributed to *B. bassiana* survival under starvation stress. Cultured in SDB broth (a nutrient-rich medium), the mycelia showed numerous punctate signals. However, once under starvation stress, the GFP signals were translocated into the mycelial vacuoles (Fig. 2A).

On water agar plates (i.e., nutrient-deficient conditions), all autophagy-deficient mutants showed a significantly reduced germination percentage (~20%); however, the wide type and complementation strains had a germination percentage of approximately 50%. On the contrary, no significant difference in the germination rate of any strain on SDAY plates (a nutrient-rich medium) was seen, with a germination percentage above 95% (Fig. 2B). In addition, the germlings of the wild type and complementation strains were obviously longer than that of the autophagy-null mutants. These differences persisted on the water agar plate and on locust hind wings (see Supplementary Fig. S4). This suggests that *BbATG1* and *BbATG8* are both involved in conidial germination under oligotrophic conditions, but are not necessary for germination in nutrient-rich conditions.

### *BbATG1* and *BbATG8* have divergent roles in spore development

**Figure 3:**
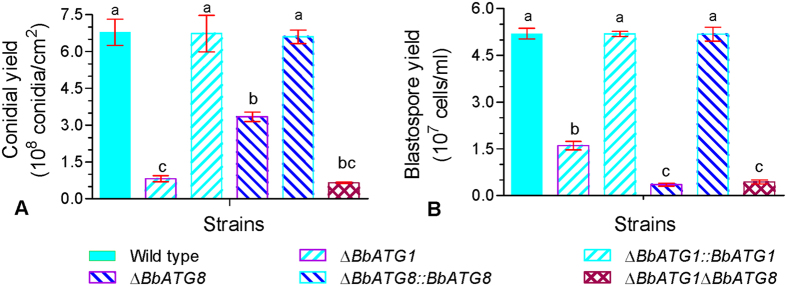
Different effects of *ATG1* and *ATG8* genes on spore development of *B. bassiana*. (**A**) Quantification of conidial yields in various strains. The indicated strain was grown on SDAY plates, and conidial production was examined at 7 days post inoculation. (**B**) Quantification of blastospore yields in various strains. Conidia of the indicated strain were inoculated into SDB media, and the blastospore production was quantified after a 3-day incubation with aeration. Significant differences are indicated by the different lowercase letters on columns. Error bars represent standard deviation from three replicate assays.

Both *BbATG1* and *BbATG8* genes were required for the production of aerial conidia and submerged blastospores. When compared with the wild type and complementation mutant strains, all autophagy-deficient mutants exhibited a dramatic decrease in conidial yield on SDAY plates, with an approximate 90% and 50% reduction in Δ*BbATG1* and Δ*BbATG8* mutants, respectively. Double disruption mutants also had a reduction in conidiation of approximately 90% (Fig. 3A). In SDB media, blastospore production was also dramatically reduced in all gene-disruption mutants compared with the wild type and complemented strains. The disruption mutants of Δ*BbATG1* and Δ*BbATG8* showed a reduction of 70% and 92% in blastospore yield, respectively, and the double mutant also had a notable reduction (95%) similar to that in the Δ*BbATG8* mutant (Fig. 3B). These results indicate that disruption of the *BbATG1* in the Δ*BbATG8* mutant exacerbates the conidiation defects in the Δ*BbATG8* mutant, but does not significantly reduce the blastospore yield of the Δ*BbATG8* mutant any further.

### *BbATG1* and *BbATG8* function differently in oxidation resistance and pathogenicity

**Figure 4:**
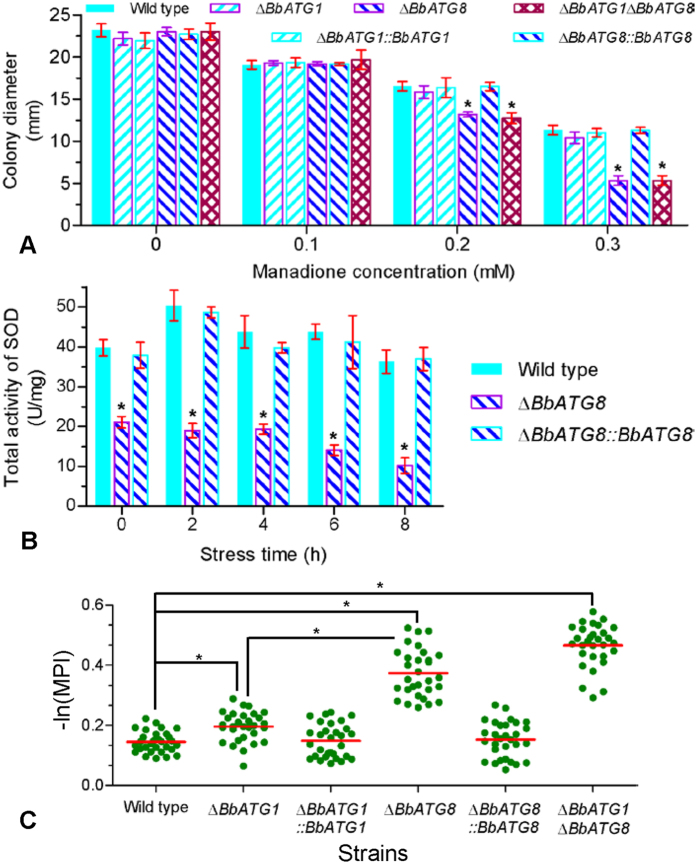
Different requirements of *ATG1* and *ATG8* genes for *B. bassiana* responses to oxidative stress. (**A**) The radial growth of indicated strains exposed to menadione stress. SDAY plate contained a gradient concentration from 0–0.3 mM menadione, and colony diameter was measured at 7 days post inoculation. (**B**) Total activities of cellular superoxide dismutases (SOD). The Δ*BbATG8* mutant strain was cultured in SDB medium for 2 d and then subjected to oxidative stress of 2 mM menadione. At the indicated time points, the mycelia were ground, and total activities of cellular soluble SOD were quantified. Asterisks on bars (in panel **A**,**B**) indicate a significant difference between the Δ*BbATG8* mutant strain and wild type or the other strains (Tukey’s honestly significant difference (HSD): *P* < 0.05). Error bars: standard deviation. (**C**) Effect of oxidative stress on hyphal bodies (*in vivo* blastospores) in haemolymph. The levels of reactive oxygen species in fungal cells were detected using a method of nitroblue tetrazolium staining. The mean pixel intensity (MPI) of cellular formazan was calculated as described in the Methods section. Statistical significance between two strains (30 cells per strain) was calculated by Mann-Whitney test and considered significant if *P* < 0.05 (indicated by an asterisk).

*BbATG1* and *BbATG8* functioned differently under oxidative stress (Fig. 4). Fungal vegetative growth was gradually inhibited with increasing concentrations of menadione. The colony diameter of all strains treated with menadione concentrations of 0–0.1 mM was not significantly different. The colony diameter of Δ*BbATG8* and the double deletion mutants was significantly different with the other four strains at menadione concentrations of 0.2 and 0.3 mM, but the growth of the Δ*BbATG1* mutant was not significantly affected (Fig. 4A). Furthermore, the Δ*BbATG8* mutants showed a significant decrease (50–70%) in overall SOD activity (Fig. 4B), but their zymograms were not significantly altered (see Supplementary Fig. S5). NBT staining showed increased formazan precipitation in the hyphal bodies of the three gene-disruption mutants (Fig. 4C; see Supplementary Fig. S6). Disruption of *BbATG1* resulted in a slight, but statistically significant increase in formazan formation compared to the wild type and complemented strains. In the Δ*BbATG8* strain, the formazan was abundantly precipitated and was slightly enhanced by the disruption of *BbATG1*.

**Figure 5:**
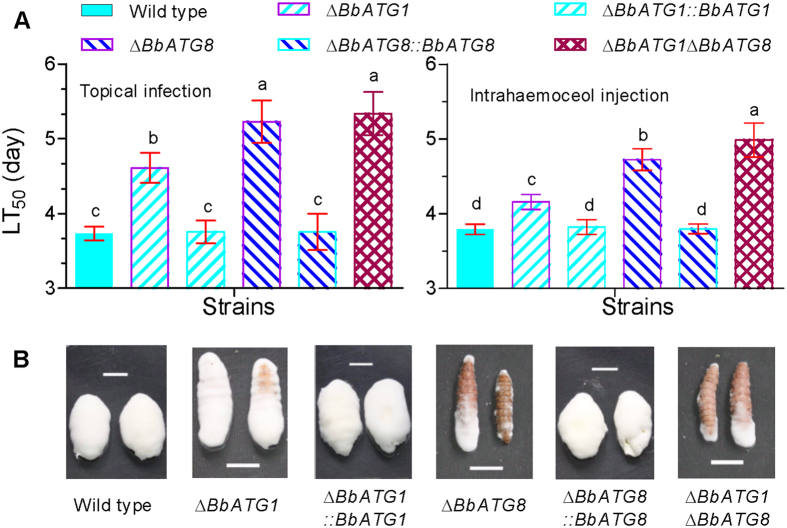
Fungal pathogenicity against bioassay insects. Conidia of the indicated strain was used to assess fungal pathogenicity with topical (**A** left) and intrahaemocoel injection (**A** right) assays, using greater wax moth, *G. mellonella* as bioassay insects. The median lethal time to mortality (LT_50_) was calculated by Probit analysis. Different letters on column bars indicate significant difference in LT_50_ between different strains (*P* < 0.05). Error bars indicate the standard deviation from three replicate assays. (**B**) Representative images showing mycosis on cadavers. Scale bar: 1 cm.

Autophagic genes *BbATG1* and *BbATG8* played different roles in fungal virulence (Fig. 5). In a cuticle penetration bioassay, the Δ*BbATG1* mutants had a slightly longer median lethal time to mortality (LT_50_), calculated as 4.61 ± 0.20 days (mean ± standard deviation) when compared with the wild type (3.73 ± 0.10) and complemented (3.75 ± 0.15) strains, with a delay of ∼25%. The LT_50_s for Δ*BbATG8* and Δ*BbATG1*Δ*BbATG8* mutants were 5.23 ± 0.28 and 5.34 ± 0.27 days, respectively, and were not significantly different (Fig. 5A left). The LT_50_ delays for Δ*BbATG8* and Δ*BbATG1*Δ*BbATG8* mutants were 40% and 43%, respectively. Based on an intrahemocoel injection assay, all of the strains killed all of the insects at 7 days post infection. The LT_50_ of the Δ*BbATG1* mutant was 4.16 ± 0.15 days, significantly different from the wild type (3.79 ± 0.07) and complemented (3.82 ± 0.10) strains, with a very slight delay of ~10% (Fig. 5A right). The Δ*BbATG8* mutant also had a longer LT_50_ (4.72 ± 0.10) which was slightly higher in the double mutant (4.99 ± 0.23). The LT_50_ delays for the Δ*BbATG8* and Δ*BbATG1*Δ*BbATG8* mutants were 24% and 32%, respectively. Additionally, ablation of *BbATG1* and *BbATG8* resulted in the impaired growth of the mycelia on host cadavers (Fig. 5B). The Δ*BbATG1* mutant generated fewer mycelia on host cadavers when compared with the wild type and complemented strains. The Δ*BbATG8* and double mutant strains were further impaired and produced only a few mycelia.

### *BbCP15* links the autophagic genes to conidiation

**Figure 6:**
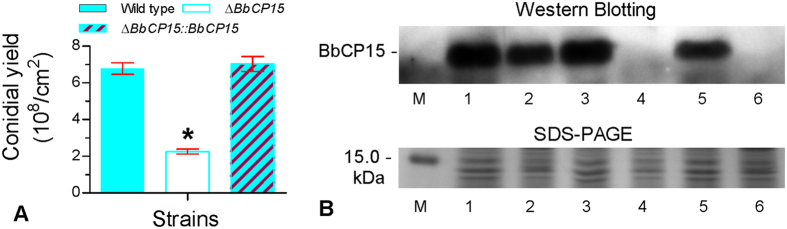
Autophagic genes, *B. bassiana ATG1* and *ATG8*, regulate conidial level of BbCP15p required for conidiation. (**A**) Conidial yield of the wild type, Δ*BbCP15* and complementation strains. SDAY plates were used to culture fungal strains, and conidial production was examined at 7 days post inoculation. An asterisk indicates a significant difference in conidial yield between the Δ*BbCP15* mutant and wild-type or complementation strains. (**B**) Western blot analysis indicated that the conidial level of BbCP15p was significantly repressed in Δ*BbATG8* mutant, and no obvious protein was detected in disruption mutants without *BbATG1* gene. Lane 1: wild type strain; lane 2: Δ*BbATG8* mutant; lane 3: Δ*BbATG8::BbATG8*; lane 4: Δ*BbATG1*; lane 5: Δ*BbATG1::BbATG1*; lane 6: Δ*BbATG1*Δ*BbATG8* and lane M: protein marker. SDS-PAGE profile of conidial proteins (30 μg) was used to show the protein integrity.

The *B. bassiana* protein BbCP15p was first isolated from conidia^28^. Gene function analysis (see Supplementary Fig. S7) indicates that *BbCP15* significantly contributes to conidial development (Fig. 6A). On SDAY plates, the conidial yield of a Δ*BbCP15* mutant strain was only approximately 30% of the wild type and complemented strains.

To investigate whether the defective conidiation in autophagy-deficient strains is related to the conidial levels of BbCP15p, western blotting was used to detect the presence of BbCP15p in conidia. The BbCP15p levels in the autophagy-deficient mutants were significantly lower than that of the wild type strain (Fig. 6B). Disruption of *BbATG8* resulted in a reduced conidial level of BbCP15p. Furthermore, no BbCP15p was detected in conidia of Δ*BbATG1* and Δ*BbATG1*Δ*BbATG8* mutants. This result suggests that reduced conidiation is related to the accumulation of BbCP15p in the conidia.

## Discussion

Autophagy is one of two major protein degradation pathways in eukaryotes^29^ and functions as an adaptive mechanism for nutrient starvation stress^2^. Increasing evidence has expanded our understanding of non-autophagic roles of autophagic genes, including signal transduction, secretory processes and cell transport^9^. In filamentous fungi, autophagy is required for conidiation, vegetative growth, fruiting-body development, life span, formation of infective structures and pathogenicity^13^,^19^,^30^,^31^,^32^,^33^,^34^,^35^. In *B. bassiana*, autophagic gene *BbATG5* is linked to spore formation and virulence^26^. This study indicates that autophagy occurs during the entire developmental cycle of *B. bassiana*. We disrupted the *ATG1* and *ATG8* genes, which are indispensable for autophagy, to explore the different roles of autophagic genes in *B. bassiana*. Disruption of these genes reduced fungal conidiation, blastospore development and virulence, and these defects were exacerbated in double disruption mutants. Only Δ*BbATG8* and double disruption mutants (but not the Δ*BbATG1* mutant) showed significantly enhanced sensitivity to oxidative stress. Additionally, we characterized the conidial protein BbCP15p, which was required for *B. bassiana* conidiation, and defects in its accumulation were related to impaired conidiation in the autophagy-null mutants.

Conidial germination is the initial step of penetration of the host cuticle for successful infection by a fungal entomopathogen^36^. Similar to the *BbATG5* gene that is required for autophagy under nutrient starvation stress^26^, both *BbATG1* and *BbATG8* are also obligatory for autophagy (see Supplementary Fig. S3). Both the *BbATG1* and *BbATG8* genes are required for conidial germination under nutrient-starvation conditions, but are not necessary for germination under nutrient-rich conditions (Fig. 2B). In *A. oryzae*, *ATG1* and *ATG8* are also required for conidial germination in absence of nutrients^13^,^14^. Additionally, the LT_50_ delay of each autophagy-null mutant was significantly greater in the topical infection assay than that in the intrahemocoel injection bioassay (Fig. 5A), which suggests that autophagy plays a significant role in the establishment of the initial infection of the host cuticle. In *M. robertsii* (another insect fungal pathogen), *MrATG1* and *MrATG8* are also required for fungal virulence. *MrATG8* regulates the formation of the appressorium, which is an important infection structure on the host cuticle^19^. However, *B. bassiana* does not produce a typical appressorium on the host’s body surface (Fig. 2C). These data suggest that *BbATG1* and *BbATG8* play a similar role in the autophagic process, which mobilizes endogenous nutrients for conidial survival and invasion on the host cuticle.

Fungal tolerance to oxidative stress is another determinant of the biocontrol potential and virulence of an entomopathogenic fungus^37^. However, *BbATG1* and *BbATG8* genes play different roles in *B. bassiana* resistance to oxidative stress. The *BbATG8* gene is essential for fungal resistance to menadione stress, but *BbATG1* is not. On the contrary, disruption mutants of the *A. niger ATG1* and *ATG8* gene are more resistant to menadione stress^38^. In the host hemocoel, the fungal cells suffer from the oxidative stress caused by the insect’s immune system^39^. It is evident that the *BbATG8* gene mediates the *B. bassiana* response to *in vivo* and *in vitro* oxidative stress and *BbATG1* does not, which suggests that the autophagic process is not significantly involved in the tolerance of *B. bassiana* to oxidative stress; however, the autophagic genes act independently in response to oxidative stress. More significantly, the *BbATG8* gene is required for maintaining 50–70% of intracellular total SOD activity under oxidative stress and normal culture conditions. Three *B. bassiana* superoxide dismutases (SOD1, SOD4 and SOD5) only account for a small part (~10%) of the intracellular SOD activity^40^,^41^. Moreover, the zymograms of SOD2 or SOD3 were not significantly affected by disrupting *BbATG8* (see Supplementary Fig. S5). This result suggests that BbATG8 has a complex effect on regulation of the superoxide dismutase activity, although the mechanism is currently unknown. However in yeast, deletion mutants of *ATG1* and *ATG8* exhibit an enhanced total SOD activity^42^. In mammal, autophagy is an important mechanism for removing damaged organelles and proteins^43^. This study does not exclude that damaged organelles and proteins are not removed by autophagy in *B. bassiana*, but *BbATG1* and *BbATG8* genes play independent roles in the regulation of antioxidant enzyme activity, which reinforces our understanding of the different roles of autophagic genes in fungal response to oxidative stress.

The dimorphic transition between hyphal and yeast-like forms is critical for the pathogenesis of mycopathogens, including mammalian, plant and insect pathogenic fungi^44^. *B. bassiana* can undergo a dimorphic transition to produce *in vitro* blastospores and hyphal bodies (*in vivo* blastospores), and these two types of cells are yeast-like^25^. Genes affecting the dimorphic transition in *B. bassiana* are associated with autophagy (e.g., *BbATG5*)^26^, the cell cycle (e.g., *BbCdc14*)^45^ and signal transduction (e.g., *BbSNF1*)^25^. *BbATG1* and *BbATG8* genes are also required for blastospore development in submerged cultures. *BbATG1* and *BbATG8* control approximately 70% and 95% of the blastospore yield in *B. bassiana*, respectively. Similarly, knockout of *BbATG5* resulted in an approximate 95% reduction in blastospore yield^26^. These results indicate that autophagic genes play different roles in regulating fungal development under submerged conditions. Various roles of *ATG1* and *ATG8* were also observed in cell budding of *U. maydis* (a plant pathogen). In this pathogen, sporidia generate new cells by budding at or near the tip of the parental cells in liquid culture, and have a very low frequency of lateral budding. *ATG1* and *ATG8* serve to maintain the normal budding sites during stationary phase, but each regulates this cellular process to a different degree^18^. The mechanisms behind the autophagic gene regulation of blastospore development are still unknown, but the reduced virulence of disruption mutants could be due, in part, to their impaired blastospore development. As a result of the impaired development in the host hemocoel, the Δ*BbATG1* mutant displays weakened mycelial growth on cadavers. The exacerbated growth defect of the Δ*BbATG8* mutant might be a combined effect of impaired blastospore development and a reduced resistance to oxidative stress.

Conidiation is not only an important reproductive process in most filamentous fungi, but also benefits fungal dispersal in a wide range of environments^46^. For entomopathogenic fungi (e.g., *B. bassiana*), conidiation determines the fungal potential of their application as biocontrol agents^47^. In filamentous fungi, many autophagic genes affecting conidiation have been functionally characterized^4^. For example, involvement of the ‘core’ autophagic genes (e.g., *ATG1*, *ATG5* and *ATG8*) in conidiation have been shown in *B. bassiana*^26^, *A. oryzae*^13^, *A. fumigatus*^15^ and *M. grisea*^16^. However, the exact mechanisms involved in the autophagic control of conidiation are still not completely known. In *M. grisea*, glycogen autophagy is required for conidiation to mobilize carbon storage. Conidiation in Δ*MgATG8* mutants could be rescued by adding carbon sources (e.g., glucose, sucrose and glucose-6-phosphate) to the culture media^48^. This result reinforces that the autophagic process is an efficient transport system in which the nutrients are transferred through tubular vacuoles^49^. Our results indicate that the *BbATG1* and *BbATG8* genes regulate the protein levels of conidial BbCP15p, which is required for *B. bassiana* conidiation. The conidiation levels were even more repressed in the Δ*BbATG1*Δ*BbATG8* mutant, suggesting that these two genes are coordinated during conidial development, although the *BbATG1* gene has a more important role than *BbATG8* gene. This study does not exclude the involvement of autophagy in nutrient recycling during *B. bassiana* conidiation; however, the BbCP15p level is significantly responsible for conidiation and is regulated by the *BbATG1* and *BbATG8* genes. Mutants without autophagic gene(s) display more severely impaired conidiation (88.0%) than Δ*BbCP15* mutants (67.0%), suggesting the possibility of other conidiation pathways under the control of these two autophagic genes. Autophagic genes regulate the expression of the conidiation-associated conidial genes, which is a new finding regarding non-autophagic roles for autophagic genes in conidial development. In filamentous fungi (e.g., *A. nidulans*), three central regulators of conidiation (i.e., *brlA*, *abaA* and *wetA*) exist^50^. In *A. oryzae*, autophagy regulates the expression of the *brlA* gene during conidiation^49^. This study suggests that the non-autophagic roles of autophagic genes might be a potential connection between autophagy and conidiation.

*B. bassiana ATG1* and *ATG8* genes contribute to conidiation by regulating the conidial levels of the conidiation-related protein BbCP15p and mediate fungal oxidation resistance by controlling total SOD activity. Our interpretation of the non-autophagic roles of *BbATG1* and *BbATG8* genes reinforces that autophagy-related proteins have comprehensive roles going beyond autophagy itself 9. In animals, protein interaction is the primary mechanism by which autophagy-related proteins perform non-autophagic functions. For example, ATG8p/LC3p functions as an important regulator of cell development by interacting with many proteins such as GTPase, GTPase-activating protein and guanine-nucleotide exchanging factors^9^. In nematodes, *UNC-51* (a homologue of yeast *ATG1*) controls axon guidance in neurons by interaction with *LET-92*, which is a catalytic subunit of protein phosphatase 2A^51^. Moreover, some autophagic genes directly regulate down-stream biological processes. For example, ATG5p is cleaved by a calcium-dependent proteinase, and its truncated form regulates cell apoptosis^14^. In the filamentous fungus *M. robertsii*, *ATG8* regulates lipid accumulation by controlling the level of a perilipin-like protein responsible for lipid storage^19^. AMP-activated protein kinase (AMPK) acts as a hub regulator of cell growth, autophagy and metabolism^52^. The AMPK protein activates autophagy by direct phosphorylation of the protein kinase ATG1p, an initiator of autophagy^53^. Functionally overlapping with autophagic genes, *B. bassiana BbSNF1* (a SNF1/AMPK ortholog) also controls the development of blastospores and conidia^25^, which suggests that the BbSNF1p protein might be a positive regulator of spore development, also probably by regulating the kinase BbATG1p. Thus, we hypothesize that signal transduction and protein interactions might mediate the non-autophagic roles of autophagy-related proteins during *B. bassiana* development and stress response. Putative proteins connecting autophagy and other physiological processes must be identified in future studies.

**Figure 7:**
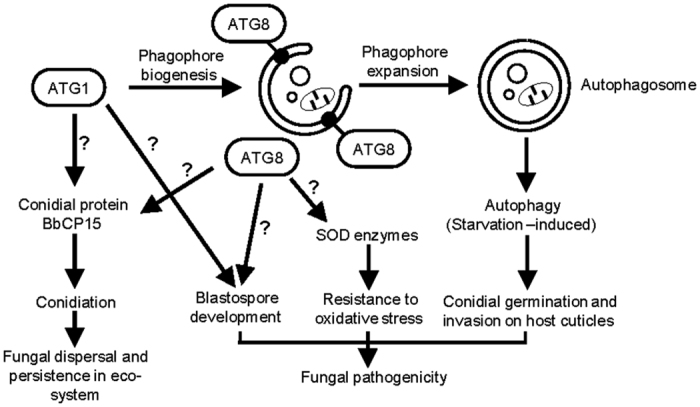
Proposed model for the autophagic and non-autophagic roles of *ATG1* and *ATG8* genes in *B. bassiana*. *BbATG1* and *BbATG8* genes have a convergent function in autophagic process required for conidial germination on host cuticles, and play different roles in spore development and resistance to oxidative stress. This initial framework was indicated by solid lines and arrows. Question marks mean that the exact mechanism in the indicated pathway needs to be clarified.

Taken all together, autophagy is constitutively associated with the entire development process of the insect pathogen *B. bassiana*. The *BbATG1* and *BbATG8* genes play a similar role in the autophagic process that is crucial for the initiation of fungal invasion of host cuticles, but have different functions during the infection cycle (Fig. 7). *B. bassiana ATG8* is essential for the fungal response to oxidative stress, while *BbATG1* is not. These two genes have different effects on fungal development and virulence, and differently affect its potential as a biocontrol agent. More significantly, we characterized *BbCP15* as a down-stream target of these two autophagic genes during conidial formation. Our studies provide an initial framework to probe the non-autophagic roles of autophagy-related proteins in the developmental and pathogenic processes of *B. bassiana*, a model insect pathogen.

## Materials and Methods

### Microbial strains and basic media

*B. bassiana* Bb2860 (wild type) was routinely grown on SDAY medium (4% glucose, 1% peptone and 1.5% agar plus 1% yeast extract). For plasmid propagation, *Escherichia coli* DH5α were cultured in Luria-Bertani medium. For fungal transformation, *Agrobacterium tumefaciens* AGL-1 acted as a T-DNA donor and was propagated in YEB medium.

### Structural and phylogenetic analysis of *BbATG1* and *BbATG8*

Sequences of ATG1p (GenBank No. EAA29429)^16^ and ATG8p (GenBank No. EAA29429)^17^ in *M. grisea* were used as the queries to search for homologs in the Bb2860 genome^27^. Additional homologs of ATG1p and ATG8p were downloaded from the NCBI database, and their phylogenetic relationship was constructed with MEGA version 5^54^.

### Visualizing autophagy in fungal development and starvation response

ATG8p is a marker for tracking the autophagic process^55^. All primers used in this study are listed in Supplementary Table S3. First, the *sur* gene cassette (conferring resistance to sulfonylurea) was amplified from the plasmid p0380-sur-gateway using the primer pair Sur-F/R, and digested with *Hind*III/*Xho*I. The resulting fragment was ligated into the same restriction sites of plasmid p0380-bar^40^, generating plasmid p0380-sur. The green fluorescent protein (GFP) gene was amplified from plasmid pABeG with the GA8-F1 and GA8-R1 primers^56^, and then fused to the 5′-end of a *BbATG8* fragment amplified with GA8-F2 and GA8-R2 primers. This hybrid fragment GFP-BbATG8 (GA8) was cloned into *Nco*I/*Bam*HI sites in plasmid pAN52-1N^57^, yielding plasmid pAN52-GA8. The GA8 expression cassette was isolated from *Pst*I-digested plasmid pAN52-GA8, and cloned into p0380-sur, generating plasmid p0380-GA8-sur. This plasmid was integrated into the Bb2860 genome, and a transformant expressing the GFP-BbATG8 was designated as strain GA8.

To visualize autophagy in aerial mycelia/conidia, conidia of strain GA8 were inoculated on SDAY plates and cultured at 25 °C. Germling and aerial mycelia were sampled at 1 and 3 days post incubation (dpi), respectively, and conidia were taken from a 7-day old culture. For fungal cells in liquid culture, conidia were inoculated into SDB (SDAY without agar) and incubated with aeration at 25 °C. The germling was isolated at 1 dpi, while mycelia and blastospores were sampled at 2 dpi. To obtain *in vivo* hyphal bodies, 5 μl of conidial suspension (10^5^ conidia/ml) was injected into the bioassay insects (*Galleria mellonella*). After 3.5 days, the hemolymph was bled and the blastospores were collected by centrifuging^25^.

To view autophagy under starvation stress, 2-day-old mycelia from SDB broth were washed with sterile water twice to remove residual nutrients and then stressed in a salt solution (CZA medium without carbon and nitrogen) for 3 h. Fluorochrome FM4-64 was used to stain vacuolar membrane. Fluorescence microscopy was performed on a confocal laser scanning microscope (LSM 710, Carl Zeiss Microscopy GmbH, Jena, Germany).

### Generation of the gene disruption and complemented strains

The genomic 5′- and 3′- flanking sequences of the *BbATG1* or *BbATG8* were amplified from the wild-type strain using the primers P_ATGx_1/2 and P_ATGx_3/4, respectively (see Supplementary Table S3; ATGx refers to *ATG1* or *ATG8*;). The resultant 5′- and 3′- fragments were digested and cloned into *Eco*RI/*Bam*HI and *Xba*I/*Spe*I sites of p0380-bar (conferring resistance to phosphinothricin)^40^, respectively, generating the disruption plasmid pKO-BbATG1 and pKO-BbATG8. The double mutant was generated by destroying *BbATG1* in a Δ*BbATG8* mutant, using the *sur* gene as the second selection marker. To generate the complementation strains, the whole gene plus the promoter were amplified with P_ATGx_7/P_ATGx_8 primers. The fragments were separately recombined into the p0380-sur-gateway plasmid^40^, generating p0380-sur-BbATG1 and p0380-sur-BbATG8, respectively. Fungal transformations were completed with *Agrobacterium*-mediated transformation methods^58^. All plasmids were first transformed into *Agrobacterium* strain, and were transformed into fungi via cocultivation of this strain with *B. bassiana* conidia. All putative mutants were first screened by PCR with the primer P_ATGx_5 and P_ATGx_6, and then confirmed by Southern blotting in which the primers P_ATGx_9 and P_ATGx_10 were used to amplify the fragment for probe preparation.

### Phenotypic assays

Phenotypic analyses such as conidial germination, spore development and virulence were performed as previously described^26^,^59^.

*Germination under starvation conditions.*  Conidial suspensions (100 μl of 10^7^ conidia/ml) were smeared onto water agar plates (1.5% agarose) and incubated for 1 day at 25 °C. SDAY plates were used as the rich medium control. Germination percentage determination and germling examination was performed by microscopy. Additionally, locust hind wing was used as a substrate to view the conidial germination pattern on insect cuticles.

*Spore development.*  Conidial suspension (100 μl of 10^6^ conidia/ml) was inoculated on SDAY plates and incubated at 25 °C. After a 7-day incubation, conidia on mycelial discs were suspended into 0.02% Tween-80. Conidial concentrations were quantified in a hemocytometer and calculated as the conidial number per square centimeter of colony.

Blastospore yield was determined in SDB medium. Conidia were inoculated into the medium at a final concentration of 10^6^ conidia/ml and cultured at 25 °C with aeration. After 3 days, the blastospore concentration was quantified using a hemocytometer and calculated as the spore number per milliliter of media.

*Fungal virulence*.  Fungal virulence was evaluated via bioassay on *G. mellonella* larvae with topical infection and intrahemocoel injection methods. In the topical application, a batch of 30–35 larvae was immersed in a conidial suspension (10^7^ conidia/ml) for 10 s, using 0.02% Tween 80 as control. For intrahemocoel injection, each larva was injected with 5 μl conidial suspension (10^5^ conidia/ml). Mortality was recorded daily and subjected to Probit analysis to determine the median lethal time (LT_50_). Cadavers from the intrahemocoel injection bioassay were incubated in moist petri dishes for 7 days, and mycosis was evaluated.

*Resistance to in vitro and in vivo oxidative stress*.  A conidial suspension (1 μl of 10^6^ conidia/ml) was spotted onto SDAY plates containing different concentrations of menadione (a superoxide radical generating reagent), ranging from 0.1–0.3 mM. The plates were incubated at 25 °C, and the colony diameters were measured at day 7.

To assess the resistance of *in vivo* hyphal bodies to oxidative stress, intracellular levels of reactive oxygen species (ROS) were reflected by formazan precipitation in cells stained with nitroblue tetrazolium (NBT). The more precipitation was formed, the more sensitive fungal cell was^60^. At 3.5 days dpi, the *in vivo* hyphal bodies were stained with NBT solution (0.5 mg/ml) for 1 h at 25 °C. The precipitation in cell was recorded via digital photography. The mean pixel intensity (MPI) of cells was quantified with Image J 1.45r software (National Institute of Health, USA). The value of pixel intensity in an image is indicated by color-gradation values ranging from zero (dark) to 255 (transparent). To normalize the MPIs, all measurements were divided by 255, and the resulting estimates were transformed by natural logarithm (ln). The negative values of the transformed estimates [–ln(MPI)] with the background (control) subtracted was used to indicate the amount of formazan precipitation.

### Functional analysis of *BbCP15* role in conidial development

BbCP15p was first identified in *B. bassiana* conidia^28^. The gene disruption strain was constructed using the same strategy as for the disruption of the autophagic genes, and all the primers used are listed in Supplementary Table S3. The conidial production was examined with the same methods reported in the Spore development section.

### Transmission electron microscopy (TEM) analysis

Autophagosomal structures in mycelia were induced and examined as previously described^26^. Two-day-old mycelia from SDB media were washed with sterile water and transferred into a salt solution. After a 3-hour induction, the mycelia were fixed overnight at 4 °C in Karnovsky’s reagent, and then overnight in 1% OsO4. The fixed mycelia were dehydrated and embedded in Spurr resin. Ultrathin sections were stained with 2% uranyl acetate followed by Reynold’s lead solution, and then examined under an H-7650 transmission electron microscope (Hitachi, Tokyo, Japan).

### Activity assay and zymogram of superoxide dismutase

The overall activity and zymogram of intracellular superoxide dismutase (SOD) were investigated as previously described^40^. Two-day-old cultures grown in SDB broth were exposed to an 8-h stress of 2 mM menadione, and mycelia were collected every 2 hours. Mycelial proteins were dissolved in 50 mM phosphate buffer (pH 7.4). The total SOD activity was determined with an SOD Assay Kit (Cat. 19160, Sigma). The protein concentration was assessed by the BCA method (Cat. KGPBCA, KeyGen Biotech). SOD activity in the protein extract was normalized as SOD units per mg protein.

To determine cellular MnSODs in the protein extract, 30 μg protein aliquots were resolved by native polyacrylamide gel electrophoresis, and the enzymes on the gel were visualized with nitroblue tetrazolium staining.

### Western blot analysis

Western blot analysis was used to detect the BbCP15p in the conidia. The protein BbCP15p was used as antigen to prepare its antibody (prepared at Zhejiang University School of Medicine, Hangzhou, China). The antiserum was used as the polyclonal antibody (anti-BbCP15)^28^. Conidial proteins were extracted with a 1% SDS solution (in a boiling bath) for 5 min. After centrifugation, the supernatant was transferred to a clean tube, and the pellet was extracted again. Conidial proteins (30 μg) were resolved in an SDS-PAGE gel (15%) and transferred to a PVDF membrane. BbCP15p was detected with anti-BbCP15 and a goat anti-rabbit IgG- horseradish peroxidase conjugate.

## Additional Information

**How to cite this article**: Ying, S.-H. *et al.* The autophagy-related genes *BbATG1* and *BbATG8* have different functions in differentiation, stress resistance and virulence of mycopathogen *Beauveria bassiana*. *Sci. Rep.* **6**, 26376; doi: 10.1038/srep26376 (2016).

## Supplementary Material

Supplementary Information
