# Supplementary material for: The autophagy-related genes BbATG1 and BbATG8 have different functions in differentiation, stress resistance and virulence of mycopathogen Beauveria bassiana
Source: Sci Rep. 2016 May 20;6:26376. doi: 10.1038/srep26376 (PMC4873834; doi:10.1038/srep26376)

Supplementary Tables and Figures

The autophagy-related genes *BbATG1* and *BbATG8* have different functions in differentiation, stress resistance and virulence of mycopathogen *Beauveria bassiana*

Sheng-Hua Ying*†, Jing Liu†, Xin-Ling Chu, Xue-Qin Xie and Ming-Guang Feng

Institute of Microbiology, College of Life Sciences, Zhejiang University, Hangzhou, Zhejiang, 310058, People's Republic of China

†: These authors contributed equally to this work.

Running title: Autophagy-related genes in entomopathogen *B. bassiana*

Keywords: *Beauveria bassiana*, autophagy-related genes, conidiation, conidial protein, blastospore development, oxidative stress, pathogenicity

***Correspondence:** Sheng-Hua Ying, Institute of Microbiology, College of Life Sciences, Zhejiang University, Hangzhou, Zhejiang, 310058, People's Republic of China; E-mail: yingsh@zju.edu.cn; Tel.: 86-571-88206199 Fax: 86-571-8820 6178

Supplementary Table S1 Domain analysis of ATG1p

	Gene ID	Species	Alignment start	Alignment end	Envelope start	Envelope end	HMM acc	HMM name	Type	HMM start	HMM end	HMM length	Bit score	E-value
**FUNGI**	A2QIL5	*Aspergillus niger*	30	334	30	336	PF00069.20	Pkinase	Domain	1	258	260	201.7	1.10E-59
	A2QIL5		649	886	646	909	PF12063.3	DUF3543	Family	5	204	238	296.4	1.10E-88
	EJP64020	*Beauveria bassiana*	25	324	23	326	PF00069.20	Pkinase	Domain	3	258	260	203.4	3.40E-60
	EJP64020		621	896	619	897	PF12063.3	DUF3543	Family	3	237	238	293.4	9.10E-88
	P53104	*Saccharomyces cerevisiae*	24	322	24	325	PF00069.20	Pkinase	Domain	1	257	260	203.4	3.20E-60
	P53104		586	893	583	894	PF12063.3	DUF3543	Family	5	237	238	214	1.70E-63
	Q4P0K0	*Ustilago maydis*	16	333	15	334	PF00069.20	Pkinase	Domain	2	259	260	188.8	9.00E-56
	Q4P0K0		708	957	707	958	PF12063.3	DUF3543	Family	2	237	238	204.3	1.50E-60
	Q52EB3	*Magnaporthe oryzae*	20	323	19	324	PF00069.20	Pkinase	Domain	2	259	260	198.6	9.60E-59
	Q52EB3		658	946	657	947	PF12063.3	DUF3543	Family	2	237	238	320.3	5.50E-96
	Q5A649	*Candida albicans*	58	352	58	354	PF00069.20	Pkinase	Domain	1	258	260	214.4	1.40E-63
	Q5A649		619	830	615	834	PF12063.3	DUF3543	Family	6	232	238	154.7	2.30E-45
	Q5BCU8	*Aspergillus nidulans*	24	329	24	332	PF00069.20	Pkinase	Domain	1	257	260	202.6	5.80E-60
	Q5BCU8		630	900	628	900	PF12063.3	DUF3543	Family	3	238	238	330.9	3.30E-99
	XP_006672480	*Cordyceps militaris*	269	588	268	590	PF00069.20	Pkinase	Domain	2	258	260	185.3	1.10E-54
	XP_006672480		864	1140	863	1141	PF12063.3	DUF3543	Family	2	237	238	287.4	6.30E-86
	XP_007819690	*Metarhizium anisopliae*	24	328	23	329	PF00069.20	Pkinase	Domain	2	259	260	198.2	1.20E-58
	XP_007819690		634	915	631	916	PF12063.3	DUF3543	Family	4	237	238	281.3	4.60E-84
**PLANT**	NP_001078281	*Arabidopsis thaliana*	20	277	20	277	PF00069.20	Pkinase	Domain	1	260	260	240.8	1.30E-71
	EEE58765	*Oryza sativa*	24	280	24	280	PF00069.20	Pkinase	Domain	1	260	260	248.6	5.40E-74
	EEE58765		631	711	554	714	PF12063.3	DUF3543	Family	157	235	238	30.1	2.50E-07

**ANIMAL**	NP_648601	*Drosophila*	11	273	9	274	PF00069.20	Pkinase	Domain	3	259	260	216.4	3.40E-64
	NP_648601	*melanogaster*	614	826	614	827	PF12063.3	DUF3543	Family	1	237	238	183.8	2.90E-54
	NP_082171		16	270	14	270	PF00069.20	Pkinase	Domain	3	260	260	234.9	7.70E-70
	NP_082171	*Mus musculus*	281	347	280	348	PF04212.13	MIT	Domain	2	68	69	71.1	5.10E-20
	NP_082171		376	444	375	444	PF04212.13	MIT	Domain	2	69	69	51.3	7.50E-14
	Q6PHR2		16	270	14	270	PF00069.20	Pkinase	Domain	3	260	260	235.6	4.70E-70
	Q6PHR2	*Homo sapiens*	281	347	280	348	PF04212.13	MIT	Domain	2	68	69	72.3	2.10E-20
	Q6PHR2		376	443	375	444	PF04212.13	MIT	Domain	2	68	69	52	4.60E-14

Supplementary Table S2 Domain analysis of ATG8p

	Gene ID	Species	Alignment start	Alignment end	Envelope start	Envelope end	HMM acc	HMM name	Type	HMM start	HMM end	HMM length	Bit score	E-value
**FUNGI**	A2QPN1	*Aspergillus niger*	13	116	13	116	PF02991.11	Atg8	Domain	1	104	104	171.7	3.00E-51
	ACJ06588	*Magnaporthe oryzae*	13	116	13	116	PF02991.11	Atg8	Domain	1	104	104	167.6	5.50E-50
	EJP69267	*Beauveria bassiana*	13	116	13	116	PF02991.11	Atg8	Domain	1	104	104	171.9	2.60E-51
	P0C075	*Candida albicans*	13	116	13	116	PF02991.11	Atg8	Domain	1	104	104	160.3	1.10E-47
	P38182	*Saccharomyces cerevisiae*	13	116	13	116	PF02991.11	Atg8	Domain	1	104	104	169.6	1.40E-50
	Q5B2U9	*Aspergillus nidulans*	13	116	13	116	PF02991.11	Atg8	Domain	1	104	104	171.1	4.40E-51
	XP_006672688	*Cordyceps militaris*	51	136	46	136	PF02991.11	Atg8	Domain	19	104	104	141.2	9.00E-42
	XP_007818863	*Metarhizium anisopliae*	13	116	13	116	PF02991.11	Atg8	Domain	1	104	104	171.9	2.60E-51
	XP_761714	*Ustilago maydis*	13	116	13	116	PF02991.11	Atg8	Domain	1	104	104	174.4	4.30E-52
**PLANT**	NP_001061171	*Oryza sativa*	14	117	14	117	PF02991.11	Atg8	Domain	1	104	104	175.3	2.20E-52
	NP_178631	*Arabidopsis thaliana*	14	117	14	117	PF02991.11	Atg8	Domain	1	104	104	173.5	8.20E-52
**ANIMAL**	NP_727447	*Drosophila melanogaster*	13	116	13	116	PF02991.11	Atg8	Domain	1	104	104	165.7	2.20E-49
	NP_009216	*Homo sapiens*	13	116	13	116	PF02991.11	Atg8	Domain	1	104	104	168.3	3.30E-50
	NP_065615	*Mus musculus*	14	116	13	116	PF02991.11	Atg8	Domain	2	104	104	165.5	2.50E-49

Supplementary Table S3 PCR primers used in this study

Primer name	Sequence (5′–3′) [a]	Restriction sites	Purpose of primer design
$P_{ATG1}1$	CCGGAATTCCGCTCCCGTCCGTCTATCTGA	*Eco*RI	Generating 5′-fragment for disruption vector
$P_{ATG1}2$	CGCGGATCCACTAATCCAAGAATATCGCCAG	*Bam*HI	
$P_{ATG1}3$	AAGCTCTAGACGCCGATTCAGTCACATACCG	*Xba*I	Generating 3′-fragment for disruption vector
$P_{ATG1}4$	GGACTAGTGCTTCGCCTGGTTATTTGCTT	*Spe*I	
$P_{ATG1}5$	GTTGAACTGCTCCGCAAGATAG	/	Screening mutants
$P_{ATG1}6$	GCTGCTCTGGGATGATTAGGC	/	
$P_{ATG1}7$	ggggACAAGTTTGTACAAAAAAGCAGGCTTAACTAAGCTTGACGGTGGCGGC	/	Obtaining the full gene for complementation
$P_{ATG1}8$	ggggACCACTTTGTACAAGAAAGCTGGGTCTGACATCGAGGCCTGTCATGCTAC	/	
$P_{ATG1}9$	CGCCGATTCAGTCACATACCG	/	Probe for Southern blotting
$P_{ATG1}10$	TGCCGAAGAGTTGTTTTGTCC	/	
$P_{ATG8}1$	CCGGAATTCGTTCTCGGTCCCATTTGTCTTG	*Eco*RI	Generating 5′-fragment for disruption vector
$P_{ATG8}2$	CGCGGATCCCATGTTGGGCAAATATGTGGTA	*Bam*HI	
$P_{ATG8}3$	AAGCTCTAGAGAGCAGCATCTACGAGGAACA	*Xba*I	Generating 3′-fragment for disruption vector
$P_{ATG8}4$	GGACTAGTCGAGACCCTGTACGCTGGAGA	*Spe*I	
$P_{ATG8}5$	GTCTTGCACCTTTCTGTACCAC	/	Screening mutants
$P_{ATG8}6$	GTGTTCTCGCCAGAGTAGGTG	/	
$P_{ATG8}7$	ggggACCACTTTGTACAAGAAAGCTGGGTGGGAGCGTGTCCAGCATTCTTC	/	Obtaining the full gene for complementation
$P_{ATG8}8$	ggggACAAGTTTGTACAAAAAAGCAGGCTTCCAGCCACCTTTTTGCCCTC	/	
		/	

$P_{ATG8}9$	CGAGAACACCTTTGGCAGCAT	/	Probe for Southern blotting
$P_{ATG8}10$	CGTATCAAACCTGCCTACAC	/	
$P_{CP}1$	AAAAAGAATTCGTGTTGGACTTCCTGACGAT	*Eco*RI	Generating 5′-fragment for disruption vector
$P_{CP}2$	AAAAAGGATCCTGTCTTACACGGGGAGTTGC	*Bam*HI	
$P_{CP}3$	AAAAATCTAGACCTCACCTTCAAAAACCTCC	*Xba*I	Generating 3′-fragment for disruption vector
$P_{CP}4$	AAAAAAGATCTTGCTTGCTTATCTAGCCTGC	*Bgl*II	
$P_{CP}5$	ACCTCGTAGTTTTAGGCAGAT	/	Screening mutants
$P_{CP}6$	ATGATTGACAAGGCTGATGG	/	
$P_{CP}7$	ggggACAAGTTTGTACAAAAAAGCAGGCTGTTGGACTTCCTGACGAT	/	Obtaining the full gene for complementation
$P_{CP}8$	ggggACCACTTTGTACAAGAAAGCTGGGTTAGCCGCCACTACCAT	/	
$P_{CP}9$	CCAACCCGAGTCTACCGCCA		Probe for Southern blotting
$P_{CP}10$	GCTCTCCGTTTTCTCATCAT		
GA8-F1	CATGCCATGGTGAGCAAGGGCGAGGAGC	*Nco*I	Fusion of GFP gene to *BbATG8*
GA8-R1	CATCCTTGAACTTGCTACGCATCTTGTACAGCTCGTCCATGCCG	/	
GA8-F2	ATGCGTAGCAAGTTCAAGGATG	/	
GA8-R2	CGCGGATCCTCAAATGCTGCCAAAGGTGTTCTC	*Bam*HI	
Sur-F	AAGAAGCCTGTCGACGTGCCAACGCCACAGTG	*Hin*dIII	Amplification of *Sur* gene
Sur-R	CATGCTCGAGGGTACCGTCGACGTGAGAGCATGCAATTC	*Xho*I-*Kpn*I	

a: The solid lines indicate the incorporated restriction sites. The sequences of *attB1* and *attB2*, required for Gateway recombination, are indicated with the dotted lines.

Supplementary figures:

Figure S1. Phylogenetic analysis of *Beauveria bassiana ATG1 and ATG8* genes. Relationships among the *ATG1* (**A**) and *ATG8* (**B**) genes were analyzed with Neighbor joining method and the bootstrap values > 50% from 1000 replicates are shown as numbers at each branch of the phylogenetic tree. GenBank accession numbers of autophagic genes are list after the abbreviations of their respective organism, which are shown as following groups, **fungi** (And: *Aspergillus nidulans*, Ang: *A. niger*, Bb: *Beauveria bassiana*, Ca: *Candida albicans*, Cm: *Cordyceps militaris*, Ma: *Metarhizium anisopliae*, Mg: *Magnaporthe grisea*, Sc: *Saccharomyces cerevisiae* and Um, *Ustilago maydis*); **plant** (At: *Arabidopsis thaliana* and Os: *Oryza sativa*); **animal** (Dm: *Drosophila melanogaster*, Hm: *Homo sapiens* and Mm: *Mus musculus*).

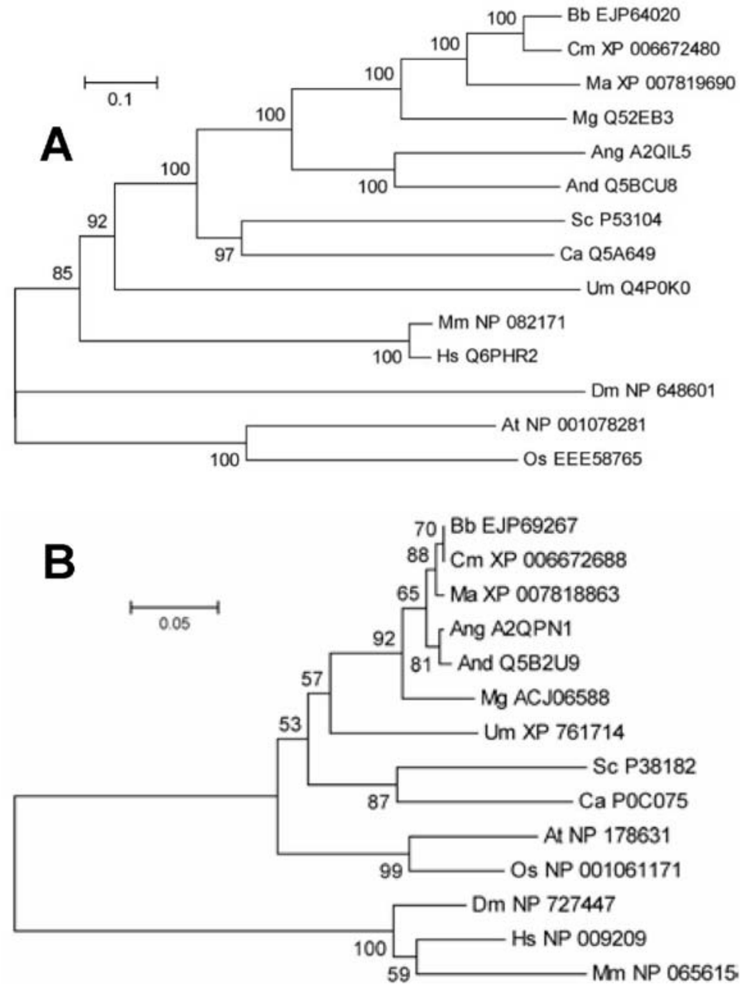

Figure S2. Molecular manipulation of *B. bassiana ATG1* and *ATG8* genes. (**A**) Schematic diagram showing gene locus, gene disruption vector, and expected homologous recombination events. ATGx refers to ATG1 or ATG8 gene, and RE represents the required restriction enzymes. (**B**) PCR screen of correct recombinant strains. Lane 1 and 4: wild type; lane 2: Δ*BbATG1* mutant; lane 3: Δ*BbATG1::BbATG1* strain; lane 5: Δ*BbATG8* mutant; lane 6: Δ*BbATG8::BbATG8* strain; lane 7: Δ*BbATG1*Δ*BbATG8* mutant and lane M: DNA molecular size standards. (**C**) Southern blotting analysis of digested genomic DNA from indicated strains. Lane 1 and 5: wild type; lane 2: Δ*BbATG1* mutant; lane 3: Δ*BbATG1::BbATG1* strain; lane 6: Δ*BbATG8* mutant; lane 7: Δ*BbATG8::BbATG8* strain; lane 4 and 8: Δ*BbATG1*Δ*BbATG8* mutant. The electrophoretic positions and sizes of the DNA fragments are shown.

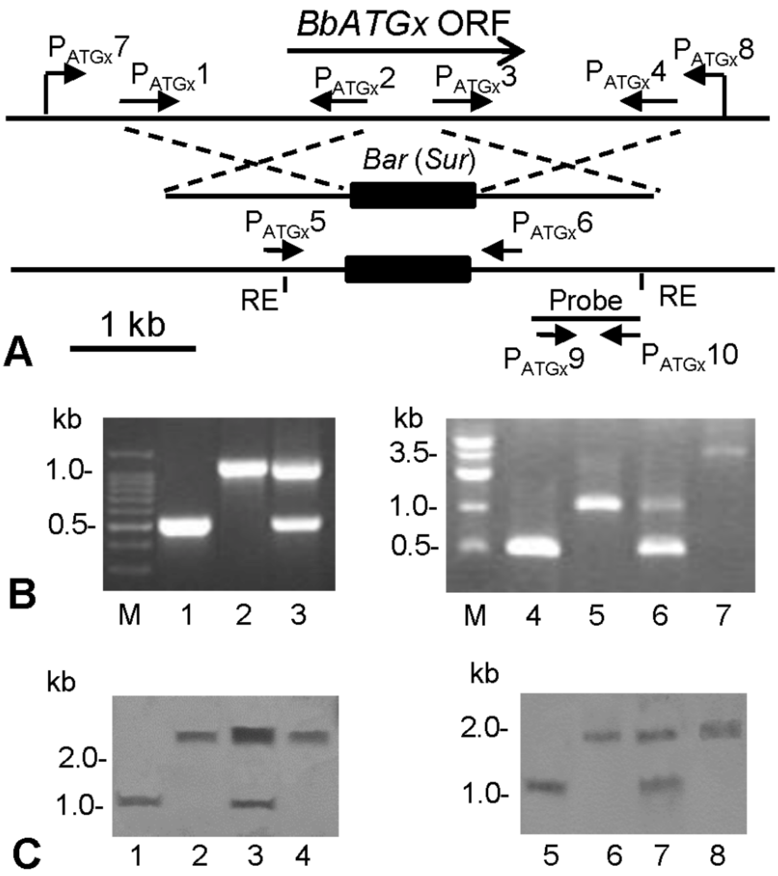

Figure S3. Gene disruption of *BbATG1* and *BbATG8* abolishes the autophagic process in *B. bassiana*. Fungal strains were incubated in SDB media for 2 days, and exposed to starvation stress in CPZ media without carbon and nitrogen sources. Transmission electron images show that the autophagic bodies were accumulated in vacuoles of wild type and complemented strains, and no obvious accumulation was found in gene disruption mutants. Scale bar represents 0.2 μm.

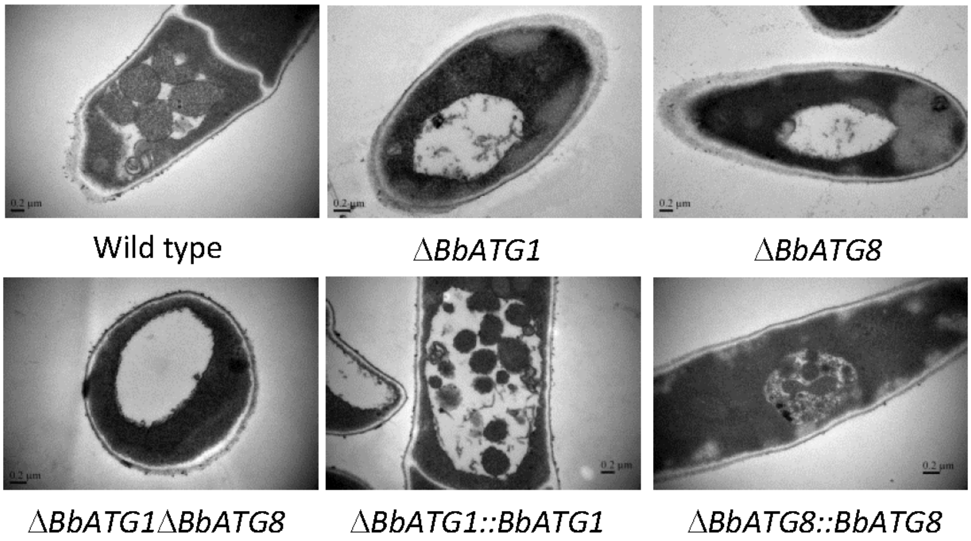

Figure S4. The patterns of conidial germination on oligotrohpic surface. Disruption of *BbATG1* and *BbATG8* genes impaired the germination ability of conidia on water agar plates (A) and host cuticles (B). Scale bars, 10 μm.

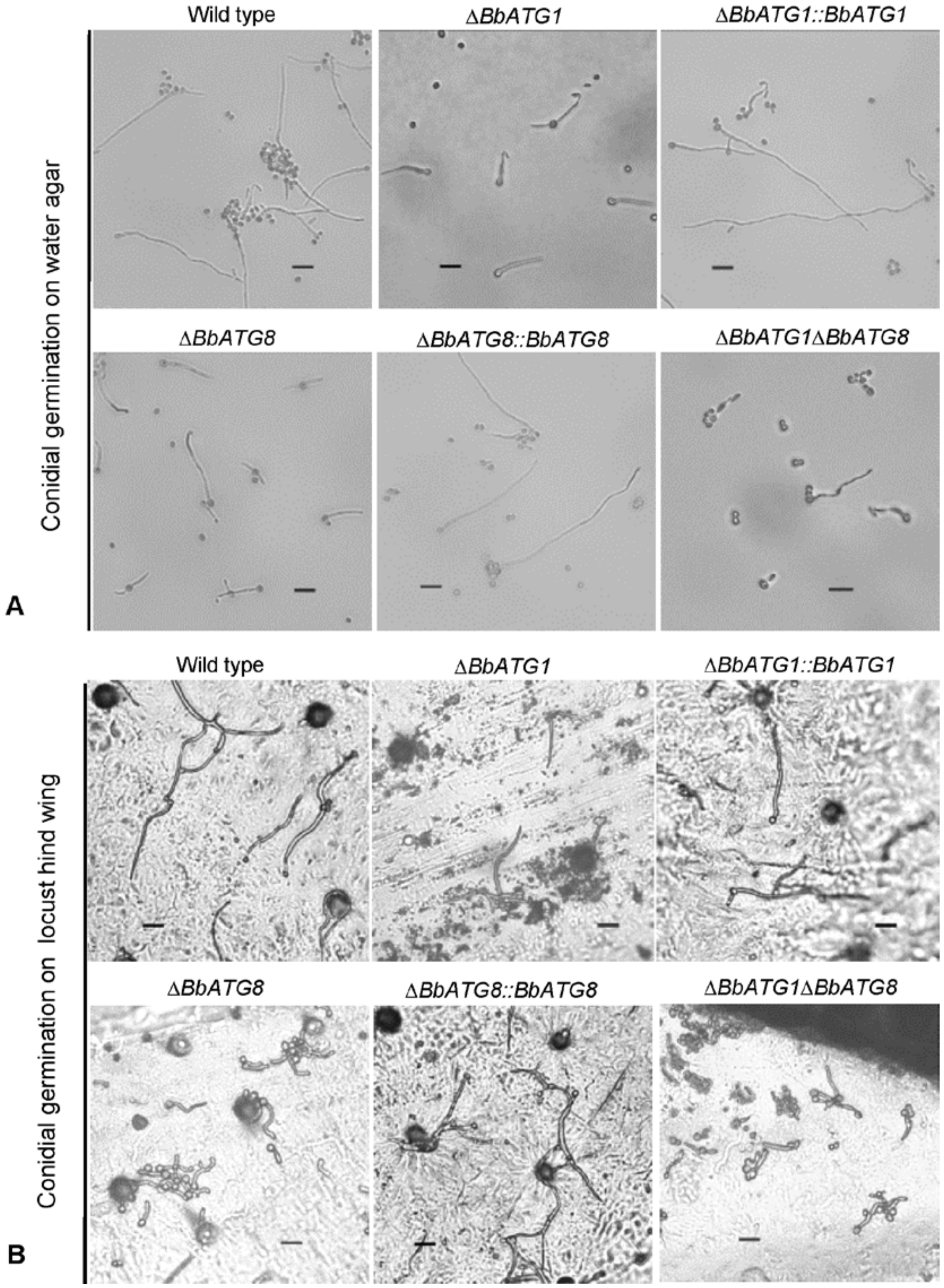

Figure S5. Zymograms for superoxide dismutases (SOD) of wild type, Δ*BbATG8* mutant and complementation strains. The 2-day-old mycelia from SDB media were exposed to an 8-h stress under 2 mM menadione, and the mycelia were sampled every 2 hours. Mycelial soluble proteins were extracted, and aliquots of 30 μg protein were resolved by native polyacrylamide gel electrophoresis. The SOD enzymes were visualized on the gel stained with nitroblue tetrazolium solution.

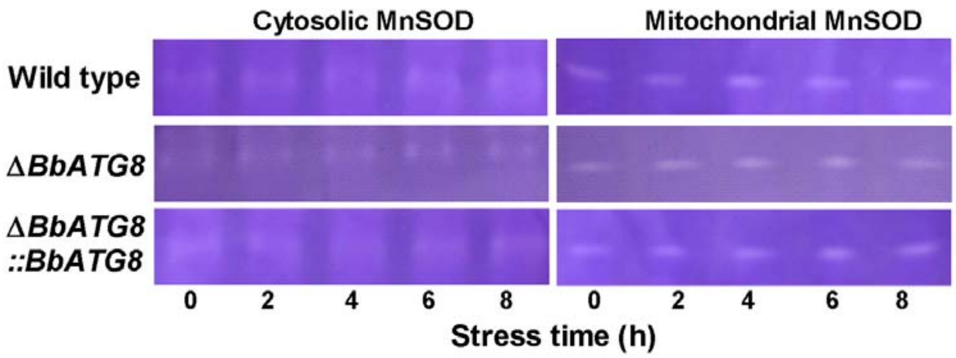

Figure S6. Representative images showing formazan precipitation in fungal cells. Hyphal bodies were harvested from the haemoceol of *Galleria mellonella* infected via injection of conidia from each indicated strain and stained with nitroblue tetrazolium. Scale bar: 5 μm.

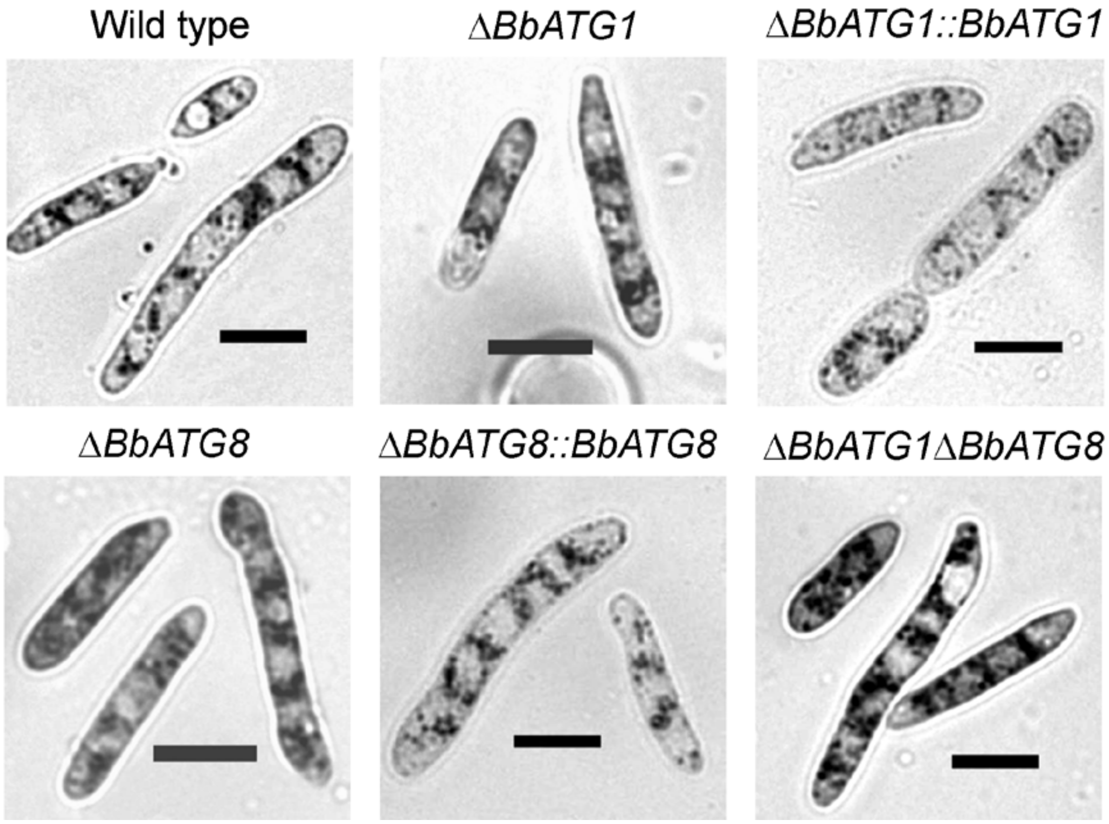

Figure S7. Gene disruption and complementation of *B. bassiana BbCP15* gene. (**A**) Schematic diagram describing the strategy of gene disruption by homologous recombination of disruption vector on the target gene locus. RE represents restriction sites for generation of DNA fragments in Southern blot. (**B**) PCR confirmation of correct recombinant strains. Lane 1: wild type; lane 2: complementation strain; lane 3: gene disruption strain and lane M: DNA molecular size standards. (**C**) Southern blotting analysis for determining correct recombination. Lane 1: wild type; lane 2: complementation strain and lane 3: gene disruption strain. The sizes of the resolved DNA fragments are shown.

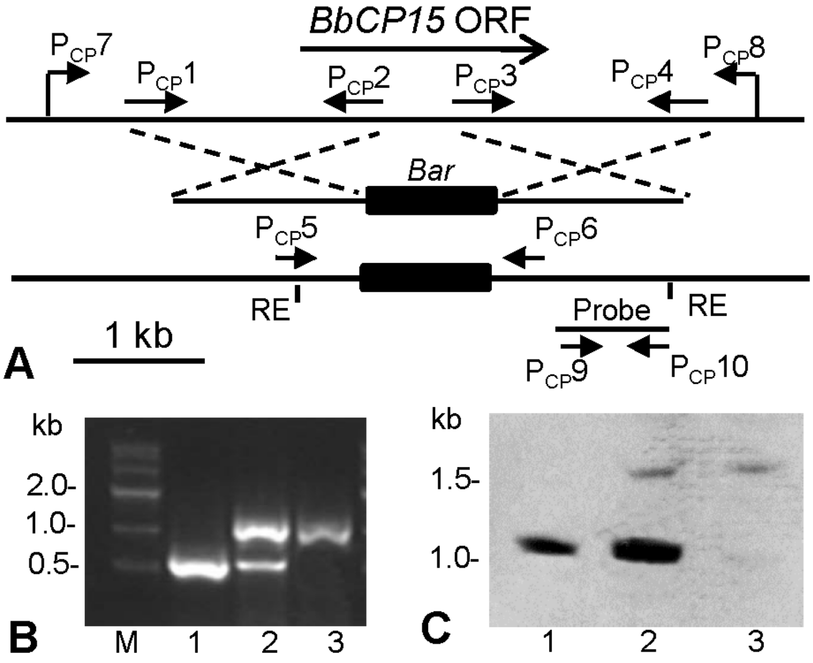

Supplement: Supplementary Information [file srep26376-s1.pdf]
